# Enhancement of antimicrobial activities of whole and sub-fractionated white tea by addition of copper (II) sulphate and vitamin C against *Staphylococcus aureus*; a mechanistic approach

**DOI:** 10.1186/1472-6882-11-115

**Published:** 2011-11-17

**Authors:** Andrew C Holloway, Simon WJ Gould, Mark D Fielder, Declan P Naughton, Alison F Kelly

**Affiliations:** 1 School of Life Sciences, Kingston University, Penrhyn Road, Kingston, London KT1 2EE, UK

## Abstract

**Background:**

Enhancement of antimicrobial plant products *e.g*. pomegranate extract by copper (II) sulphate is known. Such combinations have applications in various settings, including the identification of novel compositions to study, treat and control infection.

**Methods:**

A combination of white tea (WT) (made allowing 10 minutes infusion time at 100°C) was combined with 4.8 mM copper (II) sulphate and tested for antimicrobial effect on the viability of *Staphylococcus aureus *NCTC 06571. Comparisons were made with green (GT) and black (BT) teas. A WT sub-fraction (WTF < 1000 Da) was tested with copper (II) sulphate and 4.8 mM vitamin C. pH measurements of samples were taken for controls and to observe any changes due to tea/agent interaction. Catalase was used to investigate hydrogen peroxide release. UV-vis. was used to compare WT and WTF.

**Results:**

A 30 minute incubation at room temperature of copper (II) sulphate alone and combined with WT reduced the viability of *S. aureus *NCTC 06571 by *c.a *1 log_10 _cfu mL^-1^. GT and BT with copper (II) sulphate negated activity to buffer values. Combined with copper (II) sulphate, vitamin C, WTF and, vitamin C plus WTF all reduced the viability of *S. aureus *NCTC 06571 by *c.a*. 3.5 log_10 _cfu mL^-1^. Independent experiments showed the results were not due to pH effects. Adding WT or WTF to copper (II) sulphate resulted in increased acidity. Copper (II) sulphate alone and combined with WT required *c.a *300 μg mL^-1 ^(final concentration) catalase to restore *S. aureus *viability, WTF with copper (II) sulphate and added vitamin C required *c.a *600 μg mL^-1^. WT and WTF UV-visible spectra were similar.

**Conclusions:**

WT showed no efficacy in the combinations tested. WTF was enhanced with copper (II) sulphate and further with vitamin C. WT and WTF increased acidity of copper (II) sulphate possibly via the formation of chemical complexes. The difference in WT/WTF absorbance possibly represented substances less concentrated or absent in WTF. Investigations to establish which WTF component/s and in what proportions additives are most effective against target organisms are warranted.

## Background

Considerable efforts are being made to combat hospital and community acquired infections worldwide as well as reducing contamination within a range of settings. The incidences of both methicillin-resistant *Staphylococcus aureus *(MRSA) and other organisms *e.g*. *Clostridium difficile *demonstrate the requirement for an improved approach to preventing and rapidly controlling these and other important infections and sources of contamination [1,2]. *Staphylococcus aureus *(*S. aureus*) can cause a range of other conditions including food poisoning [3] and toxic shock syndrome [4]. Alongside enhanced hygiene and improved compliance to good practice amongst health care workers, the discovery and evaluation of new products especially within clinical settings remains a key goal for successful outbreak treatment and control [5].

In recent years, the re-emergence of classical screening approaches for natural sources of antimicrobial compounds has occurred. Numerous studies point to a range of botanicals, which includes tea, as future sources of anti-microbial agents [6-16]. This approach is driven by the potential imminent failure of current therapies against a range of resistant pathogens and by the necessity to develop low cost preparations for use in diverse geographical locations.

Tea is widely used throughout the world and has considerable applications including activity against oxidative stress, inflammation, microbes, viral infections and cancer as well as being a source of nutrients such as minerals and vitamins [13,15,17]. As anti-microbial agents, previous investigations have shown white, green and black teas (WT, GT, BT) to exhibit efficacy against a range of Gram positive and Gram negative organisms such as *S. aureus, Staphylococcus epidermidis, Streptococcus mutans *and *Escherichia coli *[15,16]. WT has been shown to contain particularly active antimicrobial components and warrants further investigation *e.g*. via sub-fraction and further physico-chemical analysis [18,19]. Tea and its constituents are also being investigated for use in clinical settings as antiseptics and as wound healing adjuncts [20]. Further studies are required to realise the potential of many natural products as sources of anti-microbial agents.

The aim of this study was to investigate the antimicrobial activity of WT which has received less attention than GT and BT. Attempts were made to enhance the activity of WT by combination with copper (II) sulphate and vitamin C as well as by sub-fractionation of WT to reduce the effect of any inhibitory substances that were present. pH measurements and UV-visible spectrometry were used to investigate possible chemical changes within mixtures as well as findings from additions of catalase to mixtures to elucidate a possible mechanism of action.

## Methods

### Microorganisms and culture conditions

*S. aureus *NCTC 06571 was grown on Nutrient agar (Oxoid, UK) prior to storage in cryogenic tubes at -80°C. Starter cultures were prepared by passaging onto nutrient agar prior to aerobic incubation overnight at 37°C; plates were subsequently stored at 5°C for one week. Fresh overnight cultures were prepared from these starter plates for use as inocula in suspension assays.

### Preparation of tea extracts and sub-fraction

Teas were prepared using boiling water extraction from 'Silver tips' WT, 'Sencha' GT and' Yorkshire' BT obtained from commercial outlets. An 8 g sample of WT, GT, or BT loose tea leaves were added to 100 mL boiling, deionised water and maintained at boiling for a period of 10 minutes. Initial volumes were restored with cold deionised water after leaves were removed prior to extracts being adjusted to pH 7 using 0.1 M NaOH. All solutions were kept in the dark and used fresh or stored at -20°C. WT sub-fraction was prepared by syringe filtration (0.45 μM and 0.1 μM Whatman) to remove particulate matter prior to filtration through a series of decreasing molecular weight cut-off ultra-centrifuge tubes (Amicon Centriprep: 30 kDa, 10 kDa, 5 kDa, 3 kDa & Pall: 1 kDa) at 3, 220 g for 60 minutes. The final filtrate was used fresh or stored in 1 mL aliquots at -20°C.

### Measurement of levels of copper found in white tea extracts

Fresh WT samples were prepared as above. Using Inductively Coupled Plasma - Atomic Emission Spectrometry (Jobin Yvon Ultima 2C ICP atomic emission spectrometer) a total of seventeen samples from batches prepared on different days were analysed to investigate their levels of copper and a mean taken.

### Antimicrobial activity of teas and WTF alone and with the addition of copper (II) sulphate and vitamin C

Fresh or frozen tea extracts were allowed to reach room temperature in the dark. *S. aureus *was suspended in Ringer's solution (Oxoid, UK) to a turbidity equivalent to 0.5 McFarland (~1.5 × 10^8 ^cfu mL^-1^). A stock solution of copper (II) sulphate in water was diluted to 4.8 mM for assays following the methodology of Stewart *et al*. [21]. A 9.6 mM vitamin C stock solution was freshly made and diluted for each experiment. For the procedures outlined below copper (II) sulphate solution was added to the tea extracts as well as Ringer's solution and left to stand for 10 minutes in the dark. An additional control of 1 mL Lambda buffer adjusted to pH 7.2 was used for all experiments [21]. A 330 μL aliquot of each tea extract was added to 700 μL of Ringer's solution and individually to the different test solutions: 4.8 mM copper (II) sulphate; 4.8 mM vitamin C; or a mixture made up 350 μL of 9.6 mM copper (II) sulphate solution with 350 μL of 9.6 mM vitamin C (the final volumes adjusted to 1 mL [21]). A 500 μL aliquot of the bacterial suspension was added and the final mixture protected from light [21].

Following 30 minutes at room temperature 150 μL was removed from the sample/inoculum mixture and added to an equal volume of 2% (v/v) Tween-80 (Sigma Chemical Co., UK) made up in Lambda buffer pH 7.2 [21]. Serial dilutions were prepared and 20 μL aliquots plated onto nutrient agar plates and incubated aerobically for 24 hours at 37°C. In addition, where necessary, volumes of 100 - 500 μL were plated to reduce the detection limit. Each assay was conducted in triplicate.

### Investigation of putative antimicrobial chemical complex formation using pH measurements

Following results of observed tea efficacies with added agents against *S. aureus *WT and WTF reaction mechanisms were investigated further by pH measurement. The pH levels of copper (II) sulphate, WT extract and WTF solutions were measured before and after combination with other additives using the same volumes as in the suspension assays, substituting Ringer's solution for the bacterial suspension. pH readings were taken following repeated calibrations with pH 4 and 7 buffers, (buffers: Thermo Electron Corporation, UK). Means of three independent samples were determined. Adjustment of pH values to 3, 5.5 (mean pH value of fresh tea) and 9 were made to extracts and to copper (II) sulphate as required using HCl and NaOH prior to undertaking viability assays.

### Investigation of differences between whole and sub-fractionated white tea using UV-visible spectrophotometry

As a means of investigating possible reasons for the difference seen in the antimicrobial activity of WTF compared to WT when combined with copper (II) sulphate the UV-vis. absorption of both extracts were compared using a Varian Cary 300 Bio UV Visible Spectrophotometer scanning between 190 - 900 nm. Mixtures were prepared as per suspensions substituting Ringers' solution for the inoculum. Mixtures were allowed to stand for 10 minutes in the dark and subsequently diluted to 25% using sterile deionised water prior to investigation. Each assay was conducted in triplicate.

### Antimicrobial activity of WT and WTF alone and with the addition of copper (II) sulphate, vitamin C and catalase

WT, WTF and combinations with copper (II) sulphate and vitamin C were prepared as above. After the 10 minute incubation, bovine liver catalase (Sigma Chemical Co., Ltd) was added to the samples to achieve a final concentration of 300, 600 or 900 μg mL^-1 ^immediately prior to the addition of the inoculum. Assays were carried out as described above.

## Results

Following a preliminary comparative study of whole WT, GT and BT against *S. aureus *NCTC 06571 all teas showed levels of viability similar to that of a buffer control. Subsequently the teas were combined with 4.8 mM copper (II) sulphate and retested. GT and BT reduced the antimicrobial efficacy of copper (II) sulphate when compared to a copper (II) sulphate control whereas WT did not. Following this WT was chosen as the best tea candidate tea to attempt enhancement of antimicrobial efficacy by alternate means.

### Levels of copper found in white tea & antimicrobial efficacy of the same and higher levels of copper (II) sulphate

Following analysis of 17 different white tea samples the levels of copper found were shown to have a range of 0.12 - 0.17 mg L^-1 ^and a mean of 0.14 mg L^-1 ^(± 0.03) similar to levels in other studies cited by Karak & Bhagat [20] (Table 1).

**Table 1:** The antimicrobial effect of different levels of copper (II) sulphate against *S. aureus *NCTC 06571 after 30 minute incubations at room temperature

Concentration of copper (II) sulphate added in assay*	Typical approximate reduction in viability following 30 minute exposure log _10 _cfu mL^-1 ^(SEM)
0 μM	N/A

0.56 μM*	N/A

1.00 μM	~0.75 (± 0.74)

4.8 mM	~2.50 (± 0.68)

9.6 mM	~3.50 (± 0.69)

N/A = not applicable as no reduction was seen. * Equivalent concentration to copper level found in whole white tea extract (0.14 mg L^-1^) by ICP-AES. SEMs shown in brackets.

When copper (II) sulphate was tested at the average copper level found in WT extract (0.14 mg L^-1 ^this being equivalent to 0.56 mM added copper (II) sulphate) there was no antibacterial effect against *S. aureus *NCTC 06571 although copper (II) sulphate tested at higher levels up to 9.6 mM showed increased antimicrobial efficacy to *c.a *3.5 log_10 _cfu mL^-1^.

### Antimicrobial activity of whole extract and a sub-fraction of white tea alone and with copper (II) sulphate and vitamin C combinations

**Figure 1:**
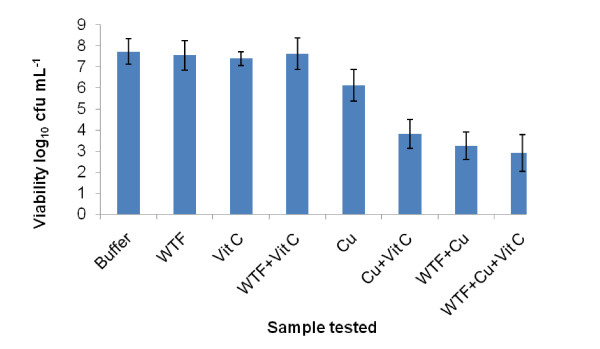
**Activities of white tea sub-fraction (< 1000 Da) alone and in different combinations with 4.8 mM copper (II) sulphate and 4.8 mM vitamin C against *S. aureus *NCTC 06571**. (WTF = white tea fraction, Vit C = vitamin C, Cu = copper (II) sulphate, '+' = combination specified. Mixtures were allowed to stand for 10 minutes before addition of culture. Sample volumes of 500 μL of Cu+Vit, WTF+Cu and WTF+Cu+Vit were used to reduce the detection limit. Bars show SEMs).

WT, WTF, vitamin C, and WTF + vitamin C did not result in a significant reduction in the viability of *S. aureus *NCTC 06571 when compared to the lambda buffer control. Whole WT in combination with 4.8 mM copper (II) sulphate and copper (II) sulphate alone both reduced the viability of *S. aureus *NCTC 06571 by *c.a*. 1 log_10 _cfu mL^-1^. The addition of 4.8 mM vitamin C to copper (II) sulphate and copper (II) sulphate to WTF resulted in reductions of *c.a*. 3.5 log_10 _cfu mL^-1 ^in both combinations. WTF with both copper (II) sulphate and added vitamin C produced a similar fall in viability (Figure 1).

### Antimicrobial activity of mixtures following addition of catalase

**Figure 2:**
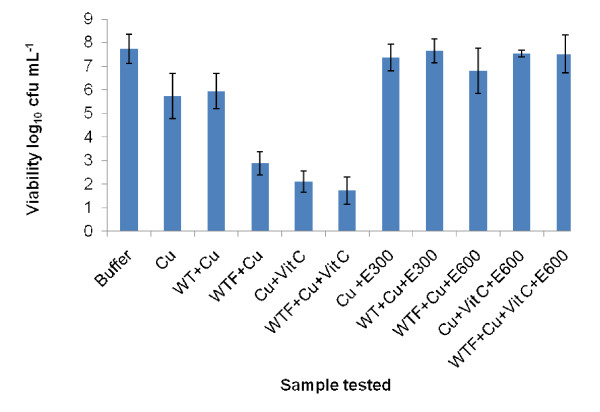
**Graph showing concentrations of catalase required to reverse antimicrobial effects of white tea sub-fraction combined with 4.8 mM copper (II) sulphate and 4.8 mM vitamin C against *S. aureus *NCTC 06571**. (Cu = copper (II) sulphate, WT = white tea, '+' = combination of agents specified, WTF = white tea fraction (< 1000 Da), Vit C = vitamin C, E300, E600 = added level of enzyme, 300 or 600 μg/mL (f.c.), Sample volumes of 500 μL of Cu+Vit C, WTF+Cu and WTF+Cu+Vit C were used to reduce the detection limit. Bars show SEMs).

The lowest concentration of bovine liver catalase able to mitigate the activity of both copper (II) sulphate and vitamin C was determined (Figure 2).

Copper (II) sulphate alone and with added WT required *c.a *300 μg/mL (final concentration; f.c.) catalase to restore the viability to control levels. The WTF with copper (II) sulphate, the vitamin C with copper (II) sulphate combination and the WTF plus vitamin C and copper (II) sulphate combinations all required *c.a*. 600 μg mL^-1 ^(f.c.) to restore viability.

### Antimicrobial activity of mixtures at differing pH

Previous investigations have shown that the adjustment of pH in the presence of metal ions can affect the size of any antimicrobial effect [22]. Therefore pH measurements of all the samples tested in the suspension assays were taken and independent experiments performed at these pH's using acid/alkali adjusted Ringer's solution to investigate the effects on *S. aureus *NCTC 06571 viability. In all cases pH was shown not to affect viability compared to a lambda buffer control (results not shown). In other experiments to investigate the effect of pH on the antimicrobial efficacy of 4.8 mM copper (II) sulphate alone no effect was seen except in the case of samples adjusted to pH 9 (using NaOH) when bactericidal activity was lost (results not shown).

### Investigation of putative antimicrobial chemical complex formation using pH measurements

**Figure 3:**
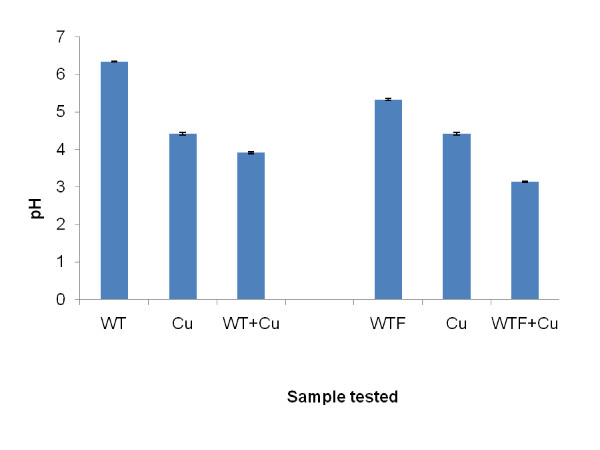
**pH measurements of whole and sub-fractionated (< 1000 Da) white tea alone and with added 4.8 mM copper (II) sulphate**. (Cu = 4.8 mM copper (II) sulphate, WT = whole white tea, '+' = combination of agents specified, WTF = white tea sub-fraction < 1000 Da. Bars show SEMs).

On addition of whole WT or WTF to 4.8 mM copper (II) sulphate the pH of resultant mixture fell by *c.a *0.5 unit (Figure 3). This fall was lower than would be expected from the simple addition of either of the two tea extracts to the copper (II) sulphate solution.

### Investigation of differences between whole and sub-fractionated white tea using UV-visible spectrophotometry

**Figure 4:**
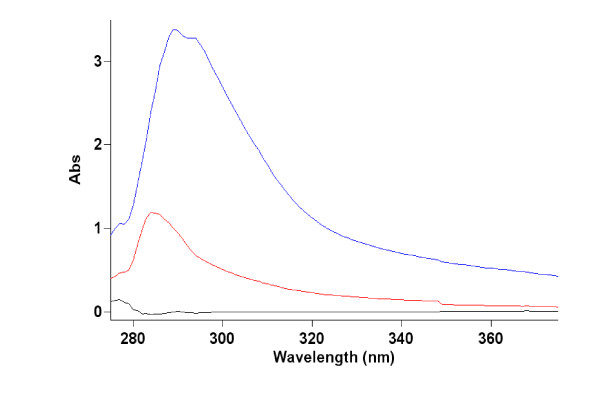
**Absorption spectra of whole white tea extract and a sub-fraction of less than 1000 Da**. Black line = water blank, Red line = white tea fraction < 1000 Da, Lilac line = whole white tea. Tea and fraction samples diluted to 25% of final concentrations used in bactericidal assays. Absorbance below 275 nm and from 375 nm to 900 nm not shown.

Whole WT showed an absorbance peak at 290 nm (Figure 4). WTF showed a lower absorption overall than whole WT with an absorption peak at *c.a *285 nm. No change in WTF absorbance was seen following addition of other agents between 190 - 900 nm (results not shown). Spectra of samples were taken at concentrations found in the original assay as well as diluted to 50% and 25%; all spectra were comparable.

## Discussion

Tea is typically made from an infusion of dried leaves or young shoots of the tea bush with hot water [23]. Different methods of leaf processing and alternative brewing methods can affect subsequent antimicrobial activity although these were not investigated these in this study [24,25].

Levels of copper found in WT following hot water extraction were found to be comparable to those reported for GT which may reflect similar methods of processing and dehydration of these two teas [20]. In BT production leaves are subject to a longer dehydrating process which could account for the higher concentration of copper reported in BT leaf product [20]. Some metal ions are also known to accumulate to varying levels in the tea bush according to cultivar type and growing conditions [26].

In suspension assays copper (II) sulphate tested at the level found in the WT extract (0.56 μM added to assay, equivalent to level of 0.14 mg L^-1 ^found in WT) was found to have the same effect as a buffer control on the viability of *S. aureus *NCTC 06571. Copper (II) sulphate tested at higher concentrations produced an increase in bactericidal effect. When WT was added to 4.8 mM copper (II) sulphate the same viability was seen as for 4.8 mM copper (II) sulphate alone (*c.a *1 log_10 _cfu mL^-1^) suggesting no additional effect on viability from the WT. When GT and BT were combined with 4.8 mM copper (II) sulphate both tea combinations showed a similar viability efficacy to buffer control levels suggesting the GT and BT had negated the bactericidal effect of the copper (II) sulphate. It is possible that GT and BT both reduced the bioavailability of the copper (II) ions by the formation of chemical complexes, which showed no antimicrobial efficacy.

Vitamin C assayed in the absence of teas enhanced the antimicrobial efficacy of copper (II) sulphate against *S. aureus *NCTC 06571 although vitamin C itself showed no antimicrobial activity when tested alone. Other investigations have shown that although vitamin C has little or no effect against many Gram positive bacteria it does enhance the bactericidal effect of copper (II) sulphate against both Gram positive and negative species [27,28]. Studies have also shown that vitamin C can further enhance the antimicrobial efficacy of plant products combined with copper (II) sulphate *e.g*. pomegranate rind extract [28-30].

WTF (< 1000 Da) was prepared to remove large molecular mass compounds suspected of inhibiting low molecular mass components with potential antimicrobial efficacy. WTF combined with 4.8 mM copper (II) sulphate produced a reduction in viability of *c.a *3.5 log_10 _cfu mL^-1 ^whereas whole WT with the same level of copper (II) sulphate produced a lower fall of *c.a *1 log_10 _cfu mL^-1^, this level in reduction in viability had been previously shown to be due to the effects of the copper (II) sulphate and not the added tea. Since whole WT was found not to affect the antimicrobial efficacy of the copper (II) sulphate it is possible that whole tea contains substances that affect the bioavailability of smaller bactericidal substances.

The antimicrobial activity of the WTF alone and with added agents against *S. aureus *NCTC 06571 is comparable to the activity found by McCarrell *et al*. who showed that another plant extract, pomegranate rind extract (PRE) which like tea contains polyphenols, did not reduce *S. aureus *NCTC 06571 viability when tested alone but was enhanced by added copper (II) sulphate and further by added vitamin C [28,29]. Comparing the results of this investigation with those of Gould *et al*. as well as McCarrell *et al*. it appears that both WTF and PRE in combination with 4.8 mM copper (II) sulphate produce a similar reduction in viability of *c.a *3.5 - 4 log_10 _cfu mL^-1 ^against the Gram positive *S. aureus *NCTC 06571 [30,31,28].

Aspects of the reaction mechanisms of the antimicrobial agents investigated in this study can be deduced. Where samples contained copper (II) sulphate the addition of catalase reversed the bactericidal effects of these samples on *S. aureus *NCTC 06571 suggesting the main effect of the copper (II) sulphate and other added agents was via the manufacture of hydrogen peroxide. Other investigators have shown that hydrogen peroxide is bactericidal to *S. aureus *[32]. It is also known that WT contains flavonoids *e.g*. catechins some of which are known to generate hydrogen peroxide in aqueous solution and more so when copper (II) ions are also present [33,34]. In this investigation hydrogen peroxide and other reactive oxygen species (ROS) could have been produced by re-oxidation of copper (I) following reduction of the added copper (II) by chemical species present in the mixtures including *e.g*. water, vitamin C and other compounds within the WTF *e.g*. flavonoids [33,34].

To observe a complete rather than partial restoration of buffer viability levels with the addition of catalase to samples containing copper (II) ions was unexpected since other mechanisms besides the manufacture of hydrogen peroxide have been suggested for the antimicrobial action of copper (II) ions on bacterial cells [35,36] . In this investigation catalase tested on copper (II) sulphate alone required 300 μg mL^-1 ^of the enzyme to produce buffer levels of viability whereas 600 μg mL^-1 ^was needed to produce the same viability with the WTF/copper (II) combination. This finding suggests that the WTF enhanced the production of hydrogen peroxide by the copper (II) ions since WTF tested in the absence of copper (II) ions had no antimicrobial effect on the viability of *S. aureus *NCTC 06571. Addition of vitamin C to the same combination did not require further amounts of added catalase for reversal suggesting the vitamin contributed little to the generation of hydrogen peroxide when WTF was also present. When WTF was absent, a combination of copper (II) sulphate and vitamin C required the same level of catalase (600 μg mL^-1 ^f.c.) to produce buffer levels of viability suggesting the vitamin C contributed to the generation of hydrogen peroxide. Possibly the WTF and the vitamin C both interact with the copper (II) ions in a similar way *e.g*. by reducing them to copper (I) and may both work at the same reaction site. If this were so then it could explain why either agent enhanced the copper (II) ion production of hydrogen peroxide and yet did not show any summative effect on the viability of *S. aureus *NCTC 06571 when both were present with the copper (II) ions. Such a mechanism is analogous to that of competitive inhibition in enzyme kinetics.

ROS such as superoxide and hydrogen peroxide can cause the collapse of cell viability [37]. *S. aureus *NCTC 06571 defends itself against the harmful effects of ROS *e.g*. hydrogen peroxide by synthesising enzymes such as catalase to deactivate ROS usually by catabolic breakdown [38]. The amount of hydrogen peroxide released by the agents in this investigation apparently exceeded the ability of the bacterial cells to adequately defend themselves by the levels of endogenous cellular catalase.

Experiments into the possible effects of pH on the suspension assays were carried out. Following three different pH adjustments (pH values of 3, 5.5, and 9) to control samples of Ringer's solution no reduction in bacterial viability was seen. Samples were tested in the same way and those containing copper (II) sulphate adjusted to pH 9 showed no reduction in viability when compared to controls and to samples adjusted to pH 3 and pH 5.5. In the case of copper (II) sulphate samples adjusted to pH 9 the addition of NaOH was accompanied by the appearance of a thick pale blue precipitate, conceivably copper (II) hydroxide. This precipitation reaction would have reduced the bioavailabilty of the copper (II) ions reducing their mobility as well as preventing them from generating any hydrogen peroxide.

In the pH experiments it was discovered that the addition of WT and WTF to copper (II) sulphate resulted in a fall in pH greater than expected from a dilution effect alone from the weakly acidic tea extracts which suggested that proton release had taken place in each case. The aqueous copper (II) sulphate which contained mainly positively charged free copper (II) ions would have had a high affinity for the negatively-charged oxygen ions within the ionised hydroxyl groups present in some tea components such as polyphenols. Possessing such high affinity copper (II) ions would tend to displace the less positively charged hydrogen ions from the hydroxyl groups in aqueous solution resulting in a greater level of free protons and thus a lower pH as observed.

In an attempt to explain the varying antimicrobial efficacies shown by the WT and WTF when combined with additives UV-vis. was used to investigate the possible formation of chemical complexes which could account for these. Other studies on plant product polyphenols have shown increases in absorbance when transition metal ions form chemical complexes with these agents [39,40]. It is tempting to speculate that with the teas investigated here the copper (II) ions were possibly absorbed by non-polyphenols which do not absorb in the UV vis. wavelength range investigated and consequently no new UV-vis. absorbance peaks were apparent within this range. WTF showed a lower absorbance and a paler colour than the WT although peak absorbance was in a region typical of polyphenols [41]. The differences seen between the WTF and WT may be attributed to the ultra-filtration process restricting the passage of larger molecules and leaving smaller ones in the filtrate.

## Conclusions

An initial comparative study of whole teas showed that GT and BT but not WT reduced the antimicrobial efficacy of copper (II) sulphate against *S. aureus *NCTC 06571. Sub-fractionated WT extracts containing substances of small molecular size (WTF < 1000 Da) combined with copper (II) sulphate and vitamin C produced active antimicrobial mixtures against *S. aureus *NCTC 06571. Neither WTF nor vitamin C showed any antimicrobial efficacy when tested in the absence of copper (II) sulphate. Larger chemical components within the whole teas potentially reduced the antimicrobial effect of added agents by forming inactive complexes although these were not evident in UV-vis. spectra.

Addition of catalase removed any antimicrobial effect of the tested combinations suggesting hydrogen peroxide was the main bactericidal agent. pH studies indicated the antimicrobial efficacy of combinations of agents was independent of acidity levels except at *c.a *pH 9 when copper (II) ions precipitated and bactericidal activity was lost.

Acidic complexes may have been formed between WTF active components and copper (II) ions since a fall in pH accompanied the addition of WTF to the copper (II) sulphate. UV-vis. absorbance at certain wavelengths suggested that the smaller more active substances present in WTF may not be polyphenols but this was by no means certain and further investigations into the nature of these components is warranted. It is plausible that other plant products containing polyphenols *e.g*. PRE when in combination with certain additives may exert their antimicrobial effects in a similar way to WTF.

On-going investigations are underway to elucidate the antimicrobial mechanism of tea components with added agents to enhance activity against *S. aureus *NCTC 06571 and other bacteria in an attempt to determine whether the efficacy of such combinations can be enhanced by pre-treatments and optimal combination ratios. It is anticipated that parallels may be drawn between the antimicrobial effects of polyphenols within tea with those from other sources such as PRE.

## Competing interests

The authors declare that they have no competing interests.

## Authors' contributions

ACH, SWJG, MDF, DPN and AFK participated in the design of the study, AH carried out the laboratory studies and all analysed the data and wrote the paper. All authors read and approved the final manuscript.

## Pre-publication history

The pre-publication history for this paper can be accessed here:

http://www.biomedcentral.com/1472-6882/11/115/prepub
